# New Approach to Managing the COVID-19 Pandemic in a Complex Tertiary Care Medical Center in Madrid, Spain

**DOI:** 10.1017/dmp.2021.63

**Published:** 2021-03-03

**Authors:** Laura M. Artiga-Sainz, Antonio Sarria-Santamera, Gonzalo Martínez-Alés, Manuel Quintana-Díaz

**Affiliations:** 1 Service de Medecine Interne, Centre Hospitalier Universitaire Caen Normandie, Caen, France; 2 Department of Medicine, Nazarbayev University School of Medicine, Nur-Sultan, Kazakhstan; 3 Department of Epidemiology, Columbia University Mailman School of Public Health, New York, NY, USA; 4 Unidad de Gestión Apoyo COVID-19, Servicio Medicina Intensiva, Hospital Universitario La Paz, Madrid, Spain

## Abstract

The coronavirus disease 2019 (COVID-19) pandemic is putting health-care systems under unprecedented stress to accommodate unexpected numbers of patients forcing a quick re-organization. This article describes the staff management experience of a third level referral hospital in the city of Madrid, Spain, one of the cities and hospitals with the largest number of COVID-19 cases.

A newly created COVID-19-specific clinical management unit (CMU) coordinated all clinical departments and conducted real-time assessments of the availability and needs of medical staff, alongside the hospital’s general management board. The CMU was able to (i) redeploy up to 285 physicians every week to bolster medical care in COVID-19 wards and forecast medical staff requirements for the upcoming week so all departments could organize their work while coping with COVID-19 needs, (ii) overview all clinical activities conducted in a medicalized hotel, and (iii) recruit a team of roughly 90 volunteer medical students to accelerate data collection and evidence generation.

The main advantage of a CMU composed by a member of every job category—its ability to generate rapid, locally adapted responses to unexpected challenges—made it perfect for the unprecedented increase in health-care need generated by the COVID-19 pandemic.

Spain has been 1 of the countries more severely affected by the COVID-19 pandemic. On March 14, 2020, the Spanish Government introduced the Decree of State of Alarm and Health Emergency in Spain to provide a legal basis to confront the critical situation that the country was facing given the uncontrolled community transmission of coronavirus disease 2019 (COVID-19). On that date, Spain has reported 5753 cases and by that date already 136 persons have died.^1^

The unique characteristics of this pandemic and the extraordinarily high volume of cases put health-care systems under unprecedented stress to accommodate all those patients. In Spain, Madrid was one of the areas that was specifically hard hit by COVID-19, and University Hospital La Paz (HULP) was one of the clinical centers that had to provide care for those patients. COVID-19 could be viewed as a disaster whose need for care outstrips the ability to expand the capacity of the system to house and treat more patients in a staff-challenged environment quite above standard volumes with a specificity: a continuous impact on health-care systems along several days, even weeks. Surge capacity, the ability to provide quality medical care during such a sudden increase in the number of patients, is one of the most important components of a hospital for responding to emergencies and disasters.

The objective of this work is to describe the response of HULP to provide the best medical care to COVID-19 patients and the crucial role that the clinical management unit (CMU), which was created amid the pandemic, played in coordinating the dynamic response of HULP in the face of the avalanche of cases that presented daily, one of the centers that had to attend one of the highest number of patients affected by this disease.^2^

HULP is a university-affiliated tertiary-level hospital, with a catchment area of more than 500,000 people. HULP has 1268 beds, divided into 3 locations (La Paz, Carlos III, Cantoblanco), and a medical staff of 1158 physicians and 564 residents.^3^ Following the publication of Royal Decree 463/2020 of March 14, 2020, declaring “the state of alarm for the management of the health crisis,” nonurgent health-care activity was paralyzed and COVID-19 patient care were assigned to be managed by the staff of the emergency department, intensive care unit (ICU), internal medicine, and pneumology services.^4^ However, it was soon evident that the clinical resources of those areas were insufficient to manage the growing volume and clinical complexity of patients, which would occupy more than half of the HULP beds during the days with the greatest epidemiological pressure (689 inpatients and 139 in the ICU). The evolution of the pandemic in HULP is shown in Figure 1.

**Figure 1.**
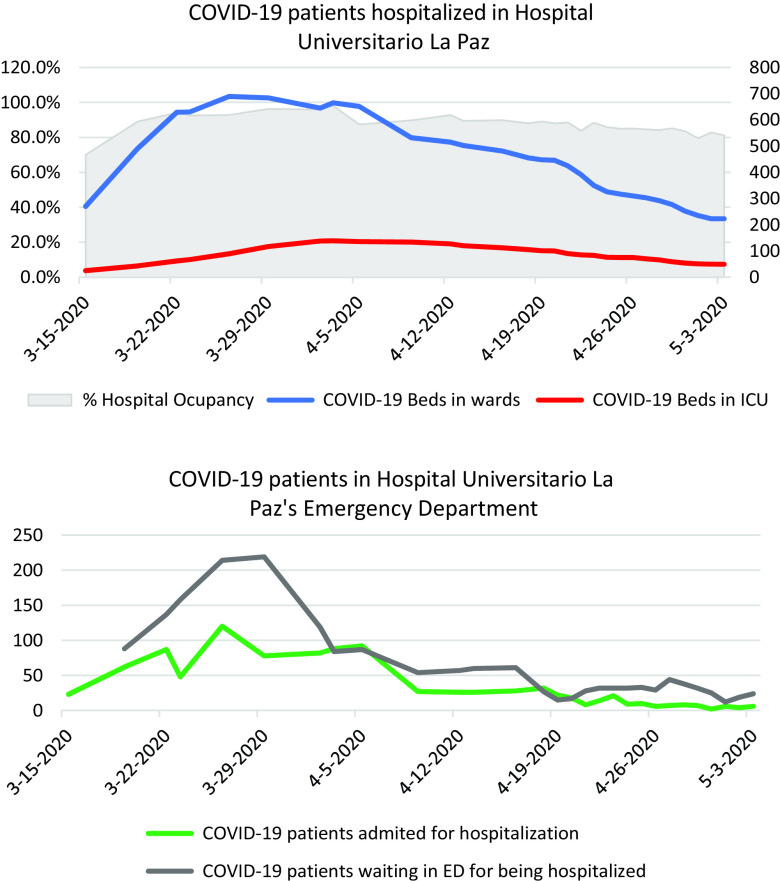
Evolution of the epidemiological pressure driven by COVID-19 patients at Hospital Universitario La Paz, Madrid, between March 15 and May 3, 2020.

The reader may be surprised both by the magnitude of resources required to treat those patients as well as for the adaptability of this complex third-level hospital to a completely new organization. All these changes were articulated in a marked redistribution of spaces, with 778 beds of the La Paz - Carlos III - Cantoblanco complex being allocated to COVID-19 areas and more than 280 doctors eventually collaborating (including both staff physicians and residents).^5^

The COVID-19 CMU was approved in March 16, 2020, and began functioning on March 23, when in HULP there were already 491 COVID-19 patients, 43 of them in ICU. The CMU was composed by an intensivist with experience in emergency management (Dr. Quintana-Díaz), the hospital chief-resident (Dr. Gutiérrez-Sancerni), her associate (Dr. Arcos-Rueda), a communication manager (Dr. Artiga-Sainz), and 1 administrative assistant (Irene Cuevas-Gordo). The role each member of the team played is expounded on Table 1. The tasks and functions of the CMU COVID-19 were:

- The management of medical personnel reallocated to provide clinical support for COVID-19 areas. Overall, up to 285 professionals from 26 medical specialties, practically from all clinical specialties at the hospital, were involved and collaborated directly or indirectly in the clinical management of COVID-19. HULP hired 50 doctors. Of these, newly recruited were 35 were “R0”; that is, medical graduates who were in the process of starting their specialty training and whose exceptional incorporation was allowed by ministerial order.^6^
- The supervision of the activity of the “Vía Castellana” medicalized hotel, carried out by 10 “R0s,” where 402 stable patients with a favorable prognosis whose outpatient follow-up was not feasible because they were not able to properly isolate at home and were admitted.
- The set-up of a data collection project of COVID-19 patients. During the first weeks of the COVID-19 wave, hospital occupancy and staff experienced compromised clinical care, making medical data collection difficult from a logistics’ point of view. The CMU decided to make a volunteering call among medical students in their 3 last years of training, given that they had both the permissions to access clinical information and the skills to provide a good-quality medical research after a quick training. Finally, 65 medical students worked daily in the project making possible to have an exhaustive database of the largest European cohort of COVID-19 patients at the end of April.^7^

**Table 1.** CSMU’s distribution of tasks and specific aspects of the job among members

Function and specific aspects of the job	Tasks
**COVID-19 Support CMU Coordinator** Assigned to an ICU specialist with experience running emergency service teams. Daily work in the critical unit allowed to have an accurate knowledge of the situation and provided interaction with doctors from different units. His link to the university as an associate professor allowed him to communicate with alumni who joined the hospital as residents who had not chosen their first allocation (R0) and students who worked with the team that gathered data.	A. Direct communication with the executive team. B. Team representation in organizational meetings on COVID-19 with the heads of internal medicine, pneumology, and emergency services. C. Coordination and handling doctor’s issues. D. Supervision of “R0” work in the medicalized hotel Vía Castellana. E. Planning the data gathering team’s activity.
**Resident Coordinator** Carried out by the head of residents or the stand-in head when she was on leave. She was, therefore, already a reference for this collective before COVID-19. Kept to her workday in the hospitalization ward in internal medicine which allowed an in-depth knowledge of the needs and immediate changes happening in this area.	A. Coordination and handling resident’s issues. B. Attendance to organizational meetings in the internal medicine ward.
**Communications Manager** Two people took on this job: the hospital’s department secretary and an “R0” with training on sanitary management. Solely dedicated to the CSMU allowing a fast response to incidents and regular checks to ensure all COVID-19 areas were functioning appropriately. They were physically located in the same area as the data gathering team, which allowed the logistical support and prompt solving of any issues this team may have had.	A. Drafting and distributing official documents. B. Coordinating general issues and handling extraordinary COVID-19 contracts. C. Support and help with the organization of the data gathering team.

This new layout of the hospital interrupted the normal activity and required medical staff to redeploy to COVID-19 areas. To understand the rapid evolution of the reorganization in HULP, we need to outline that, by March 19, when the initial redistribution of medical personnel led by the CMU started, the hospital reached a maximum occupancy of 92%, even though 122 extra beds were added to treat COVID-19 patients.

As indicated by the Hospital Management Board, the 26 COVID-19 hospitalization wards were coordinated by pulmonary and internal medicine services. In this completely ad-hoc organization, each ward had a chief coordinator (a staff physician from pulmonary or internal medicine) who was helped by 2 or 3 staff physicians of his service. In addition, depending on the size of the ward, a medical staff composed by 3-7 specialists and 2-7 residents from different medical or surgical services were allocated to each COVID-19 hospitalization ward. The workforce needed in each ward was evaluated by the coordinator together with the CMU team, first, on a daily basis, then weekly.

The emergency department also required additional support and had to be divided into a COVID-19 area and non-COVID-19 areas. Several spaces became part of the COVID-19 area, such as the gym, used by rehabilitation and physical medicine patients, some waiting rooms, and a provisional tent located in the entrance of HULP. All these sectors were coordinated by emergency staff supported by specialists and residents from different specialties.

Patients requiring critical care and mechanical ventilation could either be admitted to the ICU, postanesthesia care unit, or specific critical-labeled beds (coronary and stroke units), as the hospital had to increase critical care capacity in response to the spike in critical care demands. All critical care areas were coordinated by the physicians from the ICU and anesthesia departments, with substantial support from physicians from the cardiology and pulmonary medicine departments.

Medical staff supporting COVID-19 areas worked in 8-h shifts if deployed to the emergency department, and 8- to 12-h shifts in the rest of hospitalization wards. Most of the time, schedules were organized in weekly rotation schedules. When possible, each post remained assigned to the same service in the long term. For instance, a post in the emergency COVID-19 area had to be covered by an ophthalmology consultant all over the redeployment period.

These 7-d periods were planned to allow physicians to (i) acquire clinical experience treating COVID-19 patients while (ii) preventing clinicians from burn-out due to prolonged redeployments.

The chronological events happened as follows:

By the first week of CMU activity (March 19-26), 120 doctors (hospital staff and residents) were re-assigned to the emergency department and hospitalization wards. In this moment, a weekly redeployment plan was set forth to encourage teamwork and COVID-19 management experience, although daily readjustments on staff redeployment were made due to sick leaves and growing needs.

The “Vía Castellana” medicalized hotel started taking patients on March 23 and the first 20 “R0” were hired. To guarantee the continuity of care, staff was asked to make 8-h shifts and most of the 24-h shifts were converted into afternoon or nightly 8-h shifts.

On the second week of CMU activity (March 27 to April 3), 50 extra doctors were redeployed to hospital wards, while 26 more were assigned to the emergency department as new spaces and extra beds were set up. The following week, as COVID-19 started slowing down, 1 ward coordinated by the pneumology service was shut down. On March 26, the regional health department opened a temporary hospital in Madrid. The IFEMA Conference Centre started admitting patients to its full capacity (1500 beds), and that contributed to a significant relief on the pressure on the emergency department. This reduction of attendance in patients at the emergency department allowed the CMU to plan a more equilibrated redeployment of medical staff to partially restart other clinical areas activity.

From the 4th to the 7th week of the CMU’s Support Management Plan COVID-19 activity, HULP entered into a process for a continual readjustment of spaces to return to the old organization, and COVID-19 wards and emergency department areas were progressively closed. Concurrently, the amount of staff redeployed to ensure health-care activity in those areas was reduced always assuming a conservative approach, allocating more doctors to COVID-19 areas that what the forecast showed necessary just in case a new outbreak was to happen. An example of the chart used to distribute the medical workforce is shown in Figure 2. The CMU settled the beginning of the “new normal” in hospital activity on May 9, coinciding with the easing on lockdown measures in the country.

**Figure 2.**
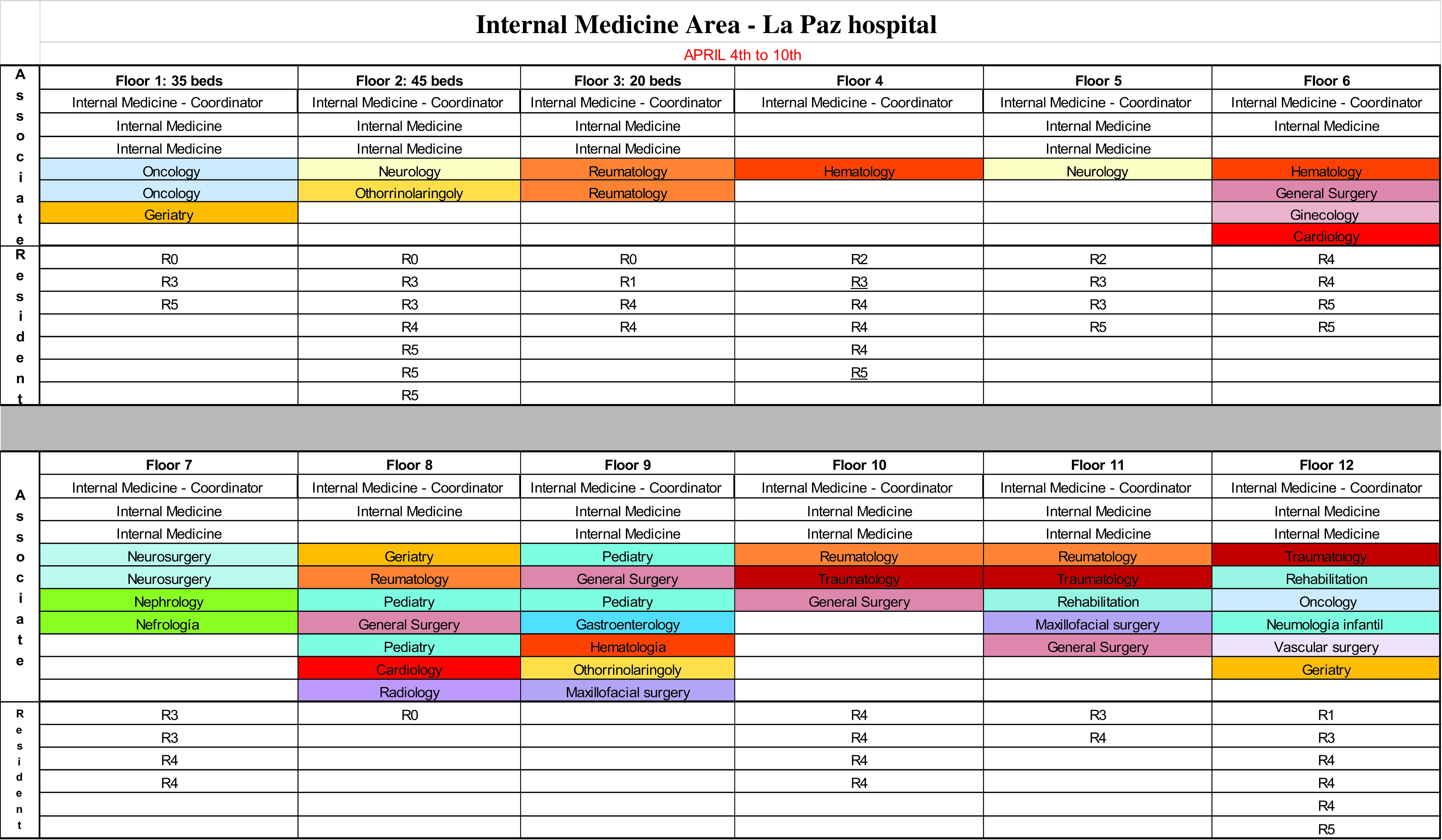
Organizational chart for the week of April 4-10 on COVID hospitalization wards coordinated by internal medicine in Hospital Universitario La Paz hospital’s main building. An equivalent chart was used in the rest of COVID-19 areas. Note that each ward workforce is composed by an internal medicine coordinator, several internal medicine associates, and 1 to 7 specialists from other services. Residents affected in hospitalization wards were mainly in the 3rd, 4th, and 5th year (R3, R4, and R5, while the younger residents were redeployed in the emergency area. The following week, a 5% reduction was expected on COVID-19 hospitalization cases, allowing to close down the smallest ward (7th floor).

In a first time, CMU redeployed medical personnel in a reactive stance, covering immediate needs and sick leaves, for the first 3 weeks. Subsequently, the experience gained managing the clinical staff together with the epidemiological forecasts available allowed the CMU to adopt a proactive perspective and design a plan for progressive withdrawal and allowing hospital activities to go back to the “new normal,” which began in the second half of April and ended on May 9, at the end of 7th week. Figure 3 shows the evolution of the reassignment of clinical personnel and hospital beds in relation to the epidemiological pressure throughout the 7 wk of the Support Management Plan COVID-19.

**Figure 3.**
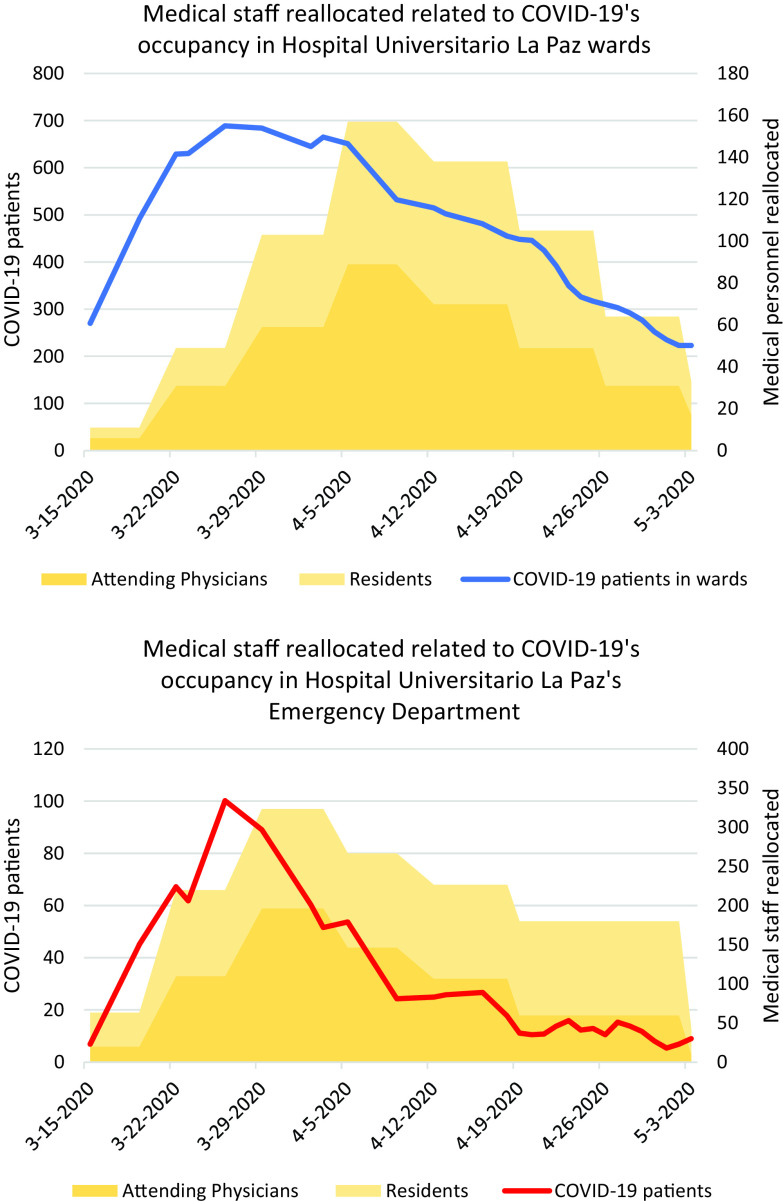
Evolution of medical staff reallocated related to COVID-19's occupancy at Hospital Universitario La Paz, Madrid, wards and emergency department.

CMU also elaborated a proposal for reorganization in case of rebound. This plan indicated the opening of a hospitalization ward for each 10% increase in COVID-19 patients when 200 patients admitted for this condition are exceeded, and the approximate assignment of a medical professional for every 4.5 patients admitted. The plan also stated the participation of doctors according to specialty and degree of training to the new COVID-19 areas.

Issues that the CMU had to face can be divided into a first phase, when a proportion of hospital beds and personnel from different clinical (eg, medical or surgical) departments were reassigned to admit COVID-19 patients, and a second phase, when these reallocated resources had to return to their usual clinical activity.

Initially, some departments had certain reluctance to devote part of their space and workforce to COVID-19 patients, which is to some extent understandable. The way to overcome this resistance was to negotiate the terms of the redeployments with those departments. In a few cases, when departments remained reticent to provide the personnel needed for COVID-19 hospital wards, the CMU was forced to ask for Hospital Management Board intervention.

Once the initial peak of the pandemic was under control, all departments requested their staff back to resume their usual clinical activity. Such requests were a source of constant negotiations between the CMU and clinical departments, as even when the peak of the pandemic was under control, the burden of COVID-19 cases greatly exceeded the usual capacity of internal medicine and pulmonology wards. These situations were most often solved by calculating staff requirements based on a realistic dimensioning of their clinical activity and using a shared decision making approach between the CMU and the departments.

For instance, if a proportion of beds and providers from the gastroenterology department were initially reassigned to admit COVID-19 patients, once the initial COVID-19 surge was under control, the CMU seized the staff needs of the department (eg, were they restarting their former activity at 50, 70, 90% of their capacity?) and planned a stepped plan where a certain proportion of the beds and staff rapidly transitioned back to former activity while others remained under COVID-19 duty for a few more days/weeks. As a result, clinical departments re-gained their usual workforce in a progressive manner that was accelerated or decelerated depending on the day-to-day COVID-19 caseload.

The organized return of medical personnel and the closure of COVID-19 areas also highlighted the relevance of a CMU, ensuring that this process took place without putting COVID-19 areas at risk of being overflowed.

The almost spontaneous constitution of a CMU to carry out personnel redeployment tasks, with a team in permanent communication selected for its proximity to the different medical groups of the hospital, allowed a rapidly adaptive work mechanic in which all the actors were involved in the different existing COVID-19 areas, guaranteeing “real-time” knowledge of the evolution of the situation. The local reorganization of staff has been positively endorsed by international guides.^8^ It should be noted that the organizational model of the CMU is considered one of the pillars of the sustainability of the National Health Service, because it provides greater efficiency and quality.^9^ Certainly, it demands a significant involvement of professionals, but in return, it renders a more comprehensive view of the patient or, in this case, of a hospital that must be rethought to respond quickly to an emerging need.

The characteristics of the Madrid metropolitan area, a densely populated zone, with an aged population having a high prevalence of hypertension or diabetes,^10^ makes it a highly vulnerable population at-risk for severe COVID-19, virtually guaranteeing a high-impact. The COVID-19 infection overwhelmed both the local primary care and hospital local infrastructure capacities, which were not experienced, trained, equipped, or resourced to support an affected casualty load of this magnitude. COVID-19 has had a tremendous death toll in Spain, but we cannot forget the heavy burden that it has had on health-care personnel: more than 54,494 infected, and 63 have died.^11^

The daily number of admissions of COVID-19 patients was increasing exponentially several days before regional and national public health authorities recognized the dimension of the COVID-19 pandemic in Spain. The hospital successfully reacted to accommodate an unprecedented number of very severe, complex, and highly infectious patients for whom scarce scientific evidence was available. Notably, these patients had to be treated by physicians with diverse clinical backgrounds—some of whom had limited experience treating severe acute respiratory distress. Under a highly stressing context and with limited time to determine the most appropriate functional and organizational approach to the management of the personnel needs driven by the pandemic, we did not consider alternatives to the one we adopted. All steps in the development of the CMU included substantial reflection and negotiation between stakeholders, which helped us redeploy personnel while keeping under consideration the preferences and needs of all clinical departments.

La Paz University Hospital is Spain’s most important major teaching hospital and has led regional and national responses to several crises, including natural disasters, aviation accidents, etc. In particular, we consider that the Hospital Management Board could react in a short period of time and adopt a CMU model because of its previous experience dealing with international health alarms: The first European case of Ebola virus in 2014 is a salient example.^12^ Following the successful response to the Ebola crisis, La Paz University Hospital keeps regular meetings and updates on emergency response protocols that were, for sure, key for the rapid coordinated response to the COVID-19 crisis.

This reality shows why surge capacity planning is critical to mass-casualty incidents like this, when thousands of persons need medical attention, and there will be insufficient resources to support the affected population if deliberate planning is not been done to address surge. The ability of our public health and health-care system to respond to catastrophic events and save as many lives as possible will remain the single most important measure of national preparedness.

The added value of the intervention carried out in the HULP lies in the adequate choice of the roles and identity of its members, and in the speed with which its constitution was executed. Although, it is still necessary to evaluate its efficiency and compare it with other approaches adopted in the field of hospital micromanagement,^13^ the HULP experience provides a practical approach on how to address the temporary restructuring of a hospital in a health emergency with mass casualties, moving from a “silo” organization toward a network-centric architecture for improved health-care response that goes beyond the normal, limited scope of clinical interest and professional demands.
